# Proximity extension assay-based targeted proteomics for biomarker discovery in adult bacterial infections

**DOI:** 10.3389/fcimb.2026.1716483

**Published:** 2026-02-13

**Authors:** Enrico Sguazzini, Carlo Montagna, Tarek Nayfeh, Martina Offer, Marta Colaneri, Francesco Petri, Cristina Banfi, Cecilia Bonazzetti, Andrea Gori, Matteo Passerini

**Affiliations:** 1 Department of Biomedical and Clinical Sciences, “L. Sacco” University Hospital, University of Milan, Milan, Italy; 2 Evidence-Based Practice Research Program, Mayo Clinic, Rochester, MN, United States; 3 Centre for Multidisciplinary Research in Health Science (MACH), University of Milan, Milan, Italy; 4 Department of Infectious Disease, Luigi Sacco Hospital, Azienda Socio Sanitaria Territoriale (ASST) Fatebenefratelli Sacco, Milan, Italy; 5 Unit of Functional Proteomics, Metabolomics and Network Analysis, Centro Cardiologico Monzino Istituto di Ricovero e Cura a Carattere Scientifico (IRCCS), Milan, Italy; 6 Department of Medical and Surgical Sciences, Alma Mater Studiorum, University of Bologna, Bologna, Italy; 7 Infectious Diseases Unit, Department for Integrated Infectious Risk Management, Istituto di Ricovero e Cura a Carattere Scientifico (IRCCS) Azienda Ospedaliero-Universitaria di Bologna, Bologna, Italy; 8 Department of Pathophysiology and Transplantation, University of Milan, Milan, Italy

**Keywords:** bacterial infections, biomarkers, immune response, PEA, proteomics, proximity extension assay, theranostics

## Abstract

**Background:**

Bacterial infections remain a major global health burden, causing significant morbidity and mortality. Despite ongoing advances, prompt diagnosis is still hampered by nonspecific host biomarkers and the inherently slow turnaround of traditional microbiological cultures. These limitations often delay the initiation of appropriate treatments. In recent years, affinity-based proteomic approaches have been explored to address this gap. Among them, the Proximity Extension Assay (PEA) has emerged as a promising multiplexed protein quantification tool, capable of simultaneously measuring hundreds of immune and inflammatory proteins with high sensitivity from minimal sample volumes. Such technologies hold the potential to identify novel biomarkers, thereby improving both diagnosis and patient management in bacterial infections.

**Main body:**

In this systematic scoping review, we examined studies applying PEA-based proteomics to adult bacterial infections. Out of the records screened, ten studies met inclusion criteria. Most were conducted in Europe and North America, relied primarily on plasma samples, and employed commercially available panels enriched for immune and inflammatory mediators. Study quality varied, with some evidence of variability and potential risk of bias. Across the 379 proteins investigated, a subset of proteins were consistently associated with bacterial infections across multiple clinical contexts, whereas others showed limited or no associations.

**Conclusions:**

Current applications of PEA-based proteomics in adult bacterial infections is limited and largely exploratory. Rather than supporting immediate clinical translation, the available evidence suggests the value of PEA-based approaches for informing biomarker discovery. Future research should prioritize well-designed, longitudinal, and pathogen-stratified studies in clinically relevant settings to strengthen evidence robustness and support the rational development of proteomics-informed diagnostic and translational strategies.

## 1 Introduction

### 1.1 Background and rationale

Bacterial infections (BIs) cause over 7 million deaths annually worldwide, mainly due to lower respiratory, bloodstream, and peritoneal or intra-abdominal infections (Ending preventable child deaths from pneumonia and diarrhoea by 2025: the integrated Global Action Plan for Pneumonia and Diarrhoea (GAPPD), 2013; Sartelli et al., 2017; Global report on the epidemiology and burden of sepsis: current evidence, identifying gaps and future directions, 2020; GBD 2019 Antimicrobial Resistance Collaborators, 2022). These diseases disproportionately affect low- and middle-income countries, making the reduction of infection-related mortality pivotal for global health equity (Rudd et al., 2020).

Biomarkers, objectively measured indicators of biological processes, are routinely employed in infectious diseases for diagnosis, staging, prognosis, and monitoring of therapeutic responses (National Cancer Institute, [[NoYear]]; Ishimine et al., 2013; Guarner et al., 2015; Mohan and Harikrishna, 2015; Ohst et al., 2018; Escadafal et al., 2020). However, traditional markers of acute-phase infection and inflammation responses often cannot distinguish BIs from viral or non-infectious inflammatory conditions (Yusa et al., 2017). This, combined with the limitations of microbiological cultures, such as long turnaround times and the inconclusive nature of negative cultures, underscores the need for more specific biomarkers (Meisner, 2005; Qu J et al., 2015).

Notably, identifying biomarkers linked to difficult-to-treat infections, especially amid rising antimicrobial resistance, can accelerate diagnosis, enable personalized treatment strategies, improve outcome prediction, and help identify subsets of patients most likely to benefit from specific therapeutic interventions (Seok and Park, 2024). Research has largely focused on sepsis, a heterogeneous syndrome with rising incidence and mortality, and lacking diagnostic gold standard (van Engelen et al., 2018). Stratifying patients based on immune responses, assessed through specific biomarkers, could improve the evaluation of targeted interventions. This is particularly relevant in syndromes where most randomized controlled trials have failed to demonstrate significant clinical benefit (Girardis et al., 2024). Yet, it is increasingly evident that no single biomarker provides sufficient diagnostic or prognostic accuracy, and that multi-marker panels may offer greater sensitivity and specificity (Kim et al., 2017; Park et al., 2022; Das et al., 2024).

These limitations highlight the need to explore alternative strategies for biomarker discovery. Among these, proteomics has emerged as a promising approach, offering dynamic and functionally relevant insights that genomic analyses alone cannot provide (Amiri-Dashatan et al., 2018). Proteins reflect the functional state of cells and tissues, making them critical for elucidating disease mechanisms and identifying clinically relevant biomarkers (D’Onofrio et al., 2022). Unlike genomic or transcriptomic approaches, proteomics directly captures downstream biological events, including post-translational modifications and dynamic changes in protein abundance that are not inferred from nucleic acid-based analyses. Proteomic research has already contributed to discovering disease-related biomarkers and increasingly aids pathogen identification, understanding disease mechanisms, and clinical diagnostics (Gobena et al., 2024). In this context, circulating plasma proteins represent particularly attractive biomarkers, as they are readily accessible, easily quantifiable, and derived from a biological matrix that is routinely collected and analyzed in clinical practice.

Within this field, affinity-based proteomics approaches, like the Proximity Extension Assay (PEA) developed by Olink Proteomics, are targeted methods enabling sensitive and high-throughput quantification of predefined proteins from minimal specimen volumes. Compared to mass spectrometry, they offer higher sensitivity for low-abundance proteins, better scalability, and improved performance in complex biological matrices (Wang et al., 2024). These features make PEA particularly well suited for biomarker discovery in infectious diseases, where subtle but clinically relevant host-response signals may present at low concentrations. PEA employs dual antibody recognition, in which matched antibody pairs, each conjugated to a unique DNA oligonucleotide, bind to target proteins. Upon proximity binding, the DNA tags hybridize and are subsequently amplified and quantified via microfluidic quantitative PCR (qPCR), enabling the relative quantification of up to 1,161 human plasma proteins from just a few microliters of biofluid (Petrera et al., 2021). This method allows for the efficient identification of biomarkers across diverse disease contexts. Furthermore, PEA technology overcomes the challenges posed by the wide dynamic range of plasma proteins, spanning over ten orders of magnitude, while offering high sensitivity, reproducibility, and the ability to measure broad protein panels with minimal specimen input (Wang et al., 2024).

### 1.2 Aim of the review

Based on these considerations, we conducted a systematic scoping review to map the current evidence on *in vivo* use of PEA-based proteomic technology (OlinkⓇ, Olink Bioscience AB, Uppsala, Sweden) in adult BIs, with the aim of characterizing how this technology has been applied in clinical research, summarizing reported proteomic findings, and identifying gaps in the literature.

## 2 Materials and methods

This scoping review was conducted in accordance with Cochrane methodology and reported following the 2020 Preferred Reporting Items for Systematic Reviews and Meta-Analyses extension for Scoping Reviews (PRISMA-ScR) (Tricco et al., 2018). The protocol was registered with PROSPERO (https://www.crd.york.ac.uk/PROSPERO/view/CRD420250651321).

### 2.1 Data sources and search strategy

A comprehensive literature search was conducted in MEDLINE, Embase (Elsevier), and Web of Science addressing the research question: How has OlinkⓇ technology been applied in the study of human BIs?. Studies published up to February 14, 2025, were included, using the search terms “Olink” AND “infection”, therefore studies employing PEA technology without explicit reference to the branded platform may not have been captured. No language or date of publication restrictions were applied.

### 2.2 Inclusion and exclusion criteria

Studies were included if they met all the following criteria: (i) *in vivo* investigations of BIs in adults, (ii) use of PEA-based proteomic analysis, and (iii) analysis conducted on any biological specimen at any stage of the disease.

Exclusion criteria comprised: (i) studies on mycobacterial infections to reduce immunopathogenic heterogeneity, (ii) studies conducted in pediatric populations, (iii) studies including patients without confirmed BIs diagnosis, (iv) studies on viral, parasitic, or fungal pathogens, (v) grey literature (e.g., conference abstracts, proceedings, preprints), and (vi) studies lacking clear stratification of clinical groups in which PEA assays were conducted.

### 2.3 Study selection

Study selection and data extraction were performed using CovidenceⓇ platform. Two independent reviewers screened retrieved records for eligibility (ES and MP), blinded to each other’s decisions. Initial screening was based on titles and abstracts, followed by full-text review. Discrepancies were resolved through discussion.

### 2.4 Data extraction

Data extraction was conducted in duplicate and included: study details (author, year of publication, country, study design), population characteristics (type of population, number of participants, clinical setting), study methodology (type of comparison group, OlinkⓇ panel or biomarkers investigated, biological specimen), and results (protein-based biomarkers reported as associated and not-associated with BIs).

### 2.5 Synthesis methods

A narrative synthesis was performed to map study characteristics and reported proteomic findings, including number of participants, biomarkers analyzed, number of analyses, type of biological specimen, and type of comparison. Protein data from individual studies were compiled into an Excel-based database. Every study compared protein expression levels between clinical groups (e.g., infected vs non-infected), and proteins exhibiting statistically significant differences in expression, as defined by the original authors, were recorded as differentially expressed proteins (DEPs). This classification reflects the reporting of primary studies and does not imply independent validation, effect size estimation, or clinical relevance assessment by the present review. We descriptively summarized the frequency with which proteins were reported as DEPs or non-DEPs across studies. Association proportion was defined as the percentage of analyses showing a statistically significant association for a given protein, out of all analyses where that protein was tested. Statistical analyses were conducted using R statistical software (version 4.4.1) and included Chi-square test, Fisher’s exact test, test of equal or given proportions and Kruskal-Wallis test, as appropriate.

### 2.6 *Post-hoc* analysis

To identify the most informative biomarkers and reduce the influence of isolated or underpowered findings, we performed a *post-hoc* sub-analysis focusing on proteins analyzed in a sufficient number of studies to allow for more reliable interpretations. To determine an appropriate threshold, we first examined the distribution of the number of analyses available for each protein across the included studies (**Supplementary Figure 1**). As most proteins were analyzed fewer than five times, we identified cut-offs aimed at excluding sparsely represented proteins while retaining those most consistently assessed across independent studies.

These proteins are hereafter referred to as high-priority proteins for the purposes of descriptive synthesis. This frequency-based strategy, in line with previous recommendations (Skates et al., 2013), was intended to descriptively focus on proteins supported by a larger number of independent observations within the existing PEA-based literature, rather than to identify proteins with greater biological or clinical relevance. The impact of applying different thresholds on data synthesis was subsequently evaluated, as detailed in the Results section.

For this subset of proteins, we retrieved biological and functional information through automated data extraction from Uniprot (protein sequence and functional annotation database, https://www.uniprot.org/) and DisGeNET (platform integrating gene-disease association data, https://disgenet.com/). The extraction process was implemented using custom R scripts and leveraging public application programming interface (API).

### 2.7 Quality assessment of studies

The risk of bias of included studies was assessed using a revised version of the ROBINS-E tool (Risk Of Bias In Non-randomized Studies of Exposures) (Higgins et al., 2024). Assessment was independently conducted by three reviewers (ES, CM, and MP), with discrepancies resolved through consensus discussion. The tool was adapted to assess the methodological quality of diagnostic biomarker studies and the results of this assessment were summarized narratively, focusing on key domains of bias: (i) selection of cases and comparison groups, (ii) comparability of study groups based on relevant baseline characteristics, and (iii) assessment of outcomes, specifically methods and protocols used for proteomic analysis.

## 3 Results

**Figure 1:**
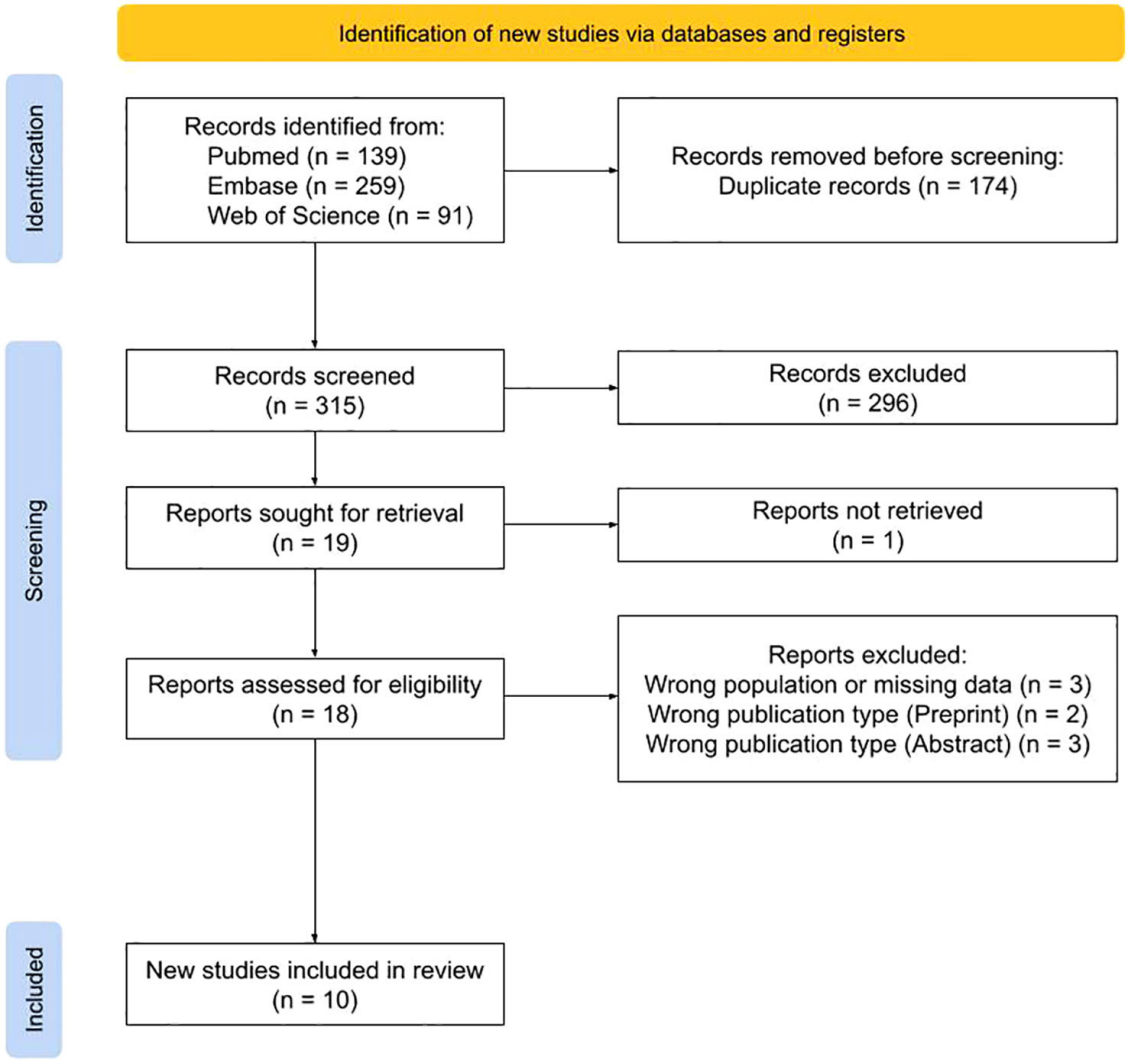
PRISMA flow diagram.

A total of 489 records were identified. After duplicate removal and title and abstract screening, 18 full-text articles were retrieved and assessed for eligibility, of which 10 met the inclusion criteria (**Figure 1**).

### 3.1 Characteristics of included studies

The included studies were conducted across diverse geographical regions, predominantly in Europe (5/10, 50%) and North America (3/10, 30%), with a smaller representation from China (2/10, 20%). Study designs encompassed case-control and cohort studies, and sample sizes varied widely (median 61.5, range 9-406).

Most studies employed commercially available biomarker panels, predominantly focusing on inflammatory and immune response proteins; only one study utilized a targeted analysis of specific markers. Regarding biological specimens, plasma was the most frequently analyzed (7/10, 70%), whereas other biological matrix types, namely serum, cerebrospinal fluid, prosthetic fluid, and synovial fluid, were each investigated in only one study.

All studies compared protein-based biomarkers in BIs versus different types of comparison groups: healthy individuals, viral infections, and other non-infectious conditions. Clinical scenarios examined spanned a broad range of infectious diseases, including community-acquired pneumonia, sepsis, meningitis, borreliosis, bloodstream infections, prosthetic joint infections (PJI), and Clostridiodes difficile infections (CDI).

Overall, included studies demonstrated substantial variability in design, specimen types, and biomarker selection, reflecting the complexity of proteomic investigations in the context of BIs (**Table 1**).

**Table 1:** Characteristics of the included studies.

Author, Year	Region	Design	Sample size	Group 1	Group(s) of comparison	Panel(s) or markers	Biological specimen	Timing of sampling
(Rischke et al., 2025)	Europe	Cohort	58	Bac CAP (n=18)	Viral CAP (n=40)	Inflammation Immune Response Organ Damage	Plasma	Baseline, Follow up
(D’Onofrio et al., 2022)	Europe	Cohort	406	Bacteremia/Bac pneumonia (n=325)	Influenza (n=81)	Inflammation	Plasma	Baseline
(Zhao et al., 2023)	China	Cohort	31	PNBM (n=15)	non-PNBM (n=16)	Immuno-Oncology	CSF	Baseline
(Fisher et al., 2022)	North America	Cohort	200	PJI (n=110)	NIAF (n=90)	Inflammation	Prosthetic fluid	Baseline
(Manasherob et al., 2024)	North America	Cohort	9	Dormant PJI* (n=3)	Non-infectious arthroplasties (n=6)	Immuno-Oncology	Plasma Synovial fluid	Baseline
(Li et al., 2023)	China	Cohort	15	Gram- ddPCR+ BSIs (n=10)	ddPCR- and Blood culture- (n=5)	Inflammation	Plasma	Baseline
(Tjernberg et al., 2023)	Europe	Case-control	127	Active LB (n=52)	Healthy blood donors (n=75)	Immune Response	Serum	Baseline
(Palma Medina et al., 2023)	Europe	Case-control	55	Bac CAP (n=21)	Bac sepsis w/o pneumonia (n=18); Healthy controls (n=16)	Inflammation Immune Response Organ Damage	Plasma	Baseline
(Rovas et al., 2022)	Europe	Cohort	65	Bac sepsis (n=43)	COVID-19 (n=22)	Inflammation Cardiovascular II	Plasma	Baseline
(Ramakrishnan et al., 2024)	North America	Case-control	89	Acute CDIs (n=59)	Healthy controls (n=30)	G-CSF, GM-CSF, IL17A	Plasma	Baseline

Bac, bacterial; CAP, community-acquired pneumonia; PNBM, post-neurosurgical bacterial meningitis; CSF, cerebrospinal fluid; PJI, prosthetic joint infection; NIAF, non-infectious arthroplasty failure; ddPCR, droplet digital PCR; BSI, bloodstream infections; LB. Lyme borreliosis; ARDS, acute respiratory distress syndrome; CDI, clostridiodes difficile infection.

*As defined by the authors, dormant PJI refers to cases resulting from direct surgical inoculation of bacteria that initially evade eradication and subsequently manifest in a delayed manner, triggered by new trauma or surgery, alterations in the host’s immune status, or quorum sensing activation.

### 3.2 Summary of findings

The proteins analyzed in each study were not always explicitly reported; however, they could be inferred from the official website of the manufacturer (https://olink.com/products/olink-target-96), based on the panels employed by the authors.

Approximately half of the analyzed proteins (207/379, 54.6%) were reported as differentially expressed in BIs compared to control groups in at least one included study. Notably, these DEPs were investigated more frequently than non-DEPs and were more often evaluated in studies comparing BIs to healthy controls or post-surgical non-infectious conditions (**Supplementary Table 1**).

### 3.3 *Post-hoc* analysis

To determine appropriate cut-offs for identifying high-priority proteins, we first examined how frequently proteins were analyzed across included studies by evaluating the number of proteins that had been analyzed once, twice, or multiple times (**Supplementary Figure 1**). The number of analyses per protein ranged from 1 to 15, with most proteins being studied fewer than five times.

Based on this distribution, we explored the impact of increasingly stringent *post-hoc* thresholds by comparing all analyzed proteins with those assessed at least five times and at least ten times (**Supplementary Figure 2**). Stricter thresholds led to a redistribution of reported associations across clinical contexts, with a progressive reduction of sparsely represented and non-associated proteins and an enrichment of proteins reported in clinically meaningful bacterial infection comparisons.

Applying the cut-off of 10 analyses, we identified 44 high-priority proteins and further characterized them based on association proportion (**Supplementary Table 3**). Most exhibited moderate association proportions, with a minority consistently associated across all comparison frameworks. Notably, fourteen proteins were recurrently reported as associated in every clinical contexts analyzed across included studies. Within this group, seven proteins, namely C-X-C motif chemokine ligand 10 (CXCL-10), interferon gamma (IFNG), TNF receptor superfamily member 9 (TNFRSF9), TNF superfamily member 14 (TNFSF14), C-C motif chemokine ligand 20 (CCL-20), C-X-C motif chemokine ligand 8 (CXCL-8), and hepatocyte growth factor (HGF), displayed an association proportion of 50. To complement these findings, we retrieved and curated functional annotations from UniProt and DisGeNET (**Table 2**). These proteins are linked to pro-inflammatory pathways, immune cell recruitment, and antimicrobial defense. Several, such as IFNG and CXCL-8, have known associations with a wide range of bacterial or mixed infectious diseases, supporting their biological plausibility as plasma biomarkers in BIs.

**Table 2:** Functional characterisation, based on UniProt and DisGeNET, of high-priority proteins that showed no association in any clinical comparison framework, followed by those that were consistently associated across all three clinical comparisons and exhibited the highest association proportions.

UniProt ID	Gene	Category	Association proportion	Function	Previously associated infectious diseases
P35225	IL-13	Non associated	0%	CK involved in allergic inflammation and anti-parasite immunity	Pneumonia, Rhinitis, Sinusitis
P60568	IL-2	Non associated	0%	CK produced by T and NK cells, key in immune response and tolerance	COVID-19, HIV, AIDS, Rhinitis, Entamoebiasis
O95760	IL-33	Non associated	0%	CK activating NF-kB and MAPK signaling	Pneumonia, Candidiasis
P05112	IL-4	Non associated	0%	CK regulating antibody production, hematopoiesis, inflammation, and effector T-cell differentiation	HCV, Pneumonia, HBV, Rhinitis, Leishmaniasis
P05113	IL-5	Non associated	0%	CK supporting eosinophil survival, differentiation, and chemotaxis	Entamoebiasis
P01137	TGFB1	Non associated	0%	Precursor of TGF-β-1, regulating immune responses and tissue repair	Pneumonia, Sepsis
P02778	CXCL-10	Associated (D’Onofrio et al., 2022; Fisher et al., 2022; Palma Medina et al., 2023; Zhao et al., 2023)	50%	Pro-inflammatory chemokine involved in chemotaxis and immune cell activation	COVID-19, HBV, Influenza
P01579	IFNG	Associated (D’Onofrio et al., 2022; Fisher et al., 2022; Palma Medina et al., 2023; Zhao et al., 2023)	50%	Type II interferon critical for antimicrobial, antiviral, and antitumor activity	HCV, Sepsis, HBV, HIV, Leishmaniasis, Pneumonia, SARS, RSV, AIDS, HSV, Infectious Arthritis, TB, Appendicitis, Entamoebiasis
Q07011	TNFRSF9	Associated (D’Onofrio et al., 2022; Fisher et al., 2022; Palma Medina et al., 2023; Zhao et al., 2023)	50%	Receptor for TNFSF9, modulating T cell activation	Arbovirus Infections
O43557	TNFSF14	Associated (D’Onofrio et al., 2022; Palma Medina et al., 2023; Zhao et al., 2023; Rischke et al., 2025)	50%	CK binding LTβR, regulating immune cell survival and inflammation	Not reported
P78556	CCL-20	Associated (D’Onofrio et al., 2022; Fisher et al., 2022; Li et al., 2023; Palma Medina et al., 2023)	50%	Chemokine ligand for CCR6, recruiting lymphocytes	Pneumonia
P10145	CXCL-8	Associated (D’Onofrio et al., 2022; Fisher et al., 2022; Li et al., 2023; Palma Medina et al., 2023)	50%	Neutrophil-attracting chemokine central to acute inflammation	UTI, Acute pyelonephritis, Leishmaniasis, Leprosy, Brucellosis, TB, Trachoma, Mycetoma, Arbovirus encephalitis
P14210	HGF	Associated (D’Onofrio et al., 2022; Fisher et al., 2022; Palma Medina et al., 2023; Rischke et al., 2025)	50%	Mitogen for hepatocytes and broad-spectrum growth factor	Polyomavirus Infections

CK, cytokine; NK, natural killer; NF-kB, nuclear factor kappa-light-chain-enhancer of activated B cells; MAPK, mitogen-activated protein kinase; HCV, hepatitis C virus; HBV, hepatitis B virus; COVID-19, coronavirus disease 2019; HIV, human immunodeficiency virus; SARS, severe acute respiratory syndrome; RSV, respiratory syncytial virus; AIDS, acquired immunodeficiency syndrome; HSV, herpes simplex virus; TB, tuberculosis; TNFSF9, TNF superfamily member 9; LTβR, lymphotoxin beta receptor; CCR6, C-C motif chemokine receptor 6; UTI, urinary tract infection.

Conversely, six proteins showed no significant associations across the included PEA-based analyses: interleukin 13 (IL-13), interleukin 2 (IL-2), interleukin 33 (IL-33), interleukin 4 (IL-4), interleukin 5 (IL-5), and transforming growth factor beta 1 (TGFB-1). These are mainly cytokines involved in regulatory or anti-parasitic immune responses, including allergic inflammation, antibody production, and eosinophil activity. Despite their established roles in immune modulation, these proteins were not annotated as associated with bacterial infectious diseases in UniProt and DisGeNET.

### 3.4 Quality of evidence

The methodological quality of the included studies was assessed using a revised version of the ROBINS-E tool. We considered three key domains: (i) selection of study population, namely diagnostic criteria and clarity of inclusion and exclusion criteria, (ii) comparability of study groups, namely adequacy of reporting baseline characteristics and matching, (iii) outcome assessment, namely clarity of proteomic assay procedures and measurement protocols.

Overall, five studies (5/10, 50%) were deemed to have low risk of bias, three (3/10, 30%) had high risk of bias, and two studies had unclear data to assess a final judgment (**Supplementary Figure 3**). While most studies clearly described the participant selection, reporting of group comparability and detailed assay protocols was often incomplete, limiting reproducibility and comparability across the included literature.

## 4 Discussion

### 4.1 Summary of main findings

Our review highlights the limited application of PEA technology in the study of BIs in adult populations. Among eligible studies, PEA was primarily used in cross-sectional designs to profile plasma protein signatures across various BIs. However, the inconsistent stratification by microbiological aetiology hinders the ability to identify pathogen-specific proteomic signatures.

Most studies used predefined commercial panels, offering standardized, reproducible platforms for broad proteomic screening. However, these restrict analyses to fixed, manufacturer-selected proteins, introducing a systemic selection bias inherent to panel design. In contrast, hypothesis-driven approaches, like Ramakrishnan et al (Ramakrishnan et al., 2024), allow tailored investigations but introduce author-dependent bias, influenced by study design, researcher’s expertise, and priori assumptions. These approaches reflect different trade-offs between breadth and focus, each carrying distinct limitations. Furthermore, the recurrence of specific proteins across studies is strongly influenced by panel composition and reuse; this should be interpreted as a feature of the current PEA research landscape rather than as independent biological or clinical validation.

Another aspect is the lack of longitudinal analyses. While Rischke et al. (Rischke et al., 2025) incorporated a longitudinal design, none explored the temporal evolution of the protein profiles during infection or antibiotic therapy. Thus, the dynamic nature of host responses to BIs remains largely unexplored within PEA literature. Overall, these findings underscore the need for more comprehensive and methodologically consistent studies, including pathogen-specific stratification and longitudinal sampling, to fully exploit PEA’s potential in BIs research.

Importantly, as a scoping review, this work aimed to map how PEA-based proteomics has been applied in adult BIs and to summarize reported findings, rather than to formally rank biomarkers or establish clinical validity. Any apparent recurrence of specific proteins across studies should therefore be interpreted as a reflection of reporting frequency within the existing literature, rather than as evidence of superior diagnostic or biological performance. Similarly, all proportions and reported associations across diverse clinical contexts and different biological matrices are descriptive and intended to map the breadth and fragmentation of PEA applications in bacterial infections.

### 4.2 Comparison with previous studies

Our analysis builds on previous literature that has highlighted the potential of proteomic approaches for biomarker discovery in infectious diseases. Earlier investigations have primarily focused on untargeted, system-wide strategies such as mass spectrometry-based proteomics (Gobena et al., 2024). This technology revealed key pathogen-host interactions, uncovering biological mechanisms underlying infectious diseases and characterizing structure and composition of both viral and bacterial pathogens (Greco and Cristea, 2017). Within biomarker research, broad-spectrum proteomic techniques are generally more suitable for the initial identification of potential candidates, whereas targeted methods are preferred for subsequent validation and clinical translation (Sobsey et al., 2020). In this regard, high-throughput multiplex immunoassays like PEA technology offer a complementary approach addressing some mass spectrometry limitations, particularly detecting low-abundance and low-molecular-weight plasma proteins (Petrera et al., 2021).

To the best of our knowledge, this study represents the first systematic investigation of the *in vivo* applications of PEA technology in BIs.

### 4.3 Strengths and limitations of the review

A major strength of our review is the inclusion of multiple databases, and the absence of restrictions on publication date or geographic setting. The methodological approach, including clearly defined selection criteria and independent quality assessment, enhances the transparency and reproducibility of our findings. Additionally, the integration of functional annotations from UniProt and DisGeNET allowed us to functionally contextualize the high-priority proteins identified.

Nonetheless, some limitations must be acknowledged. First, the significant heterogeneity in study designs, patient populations, biological specimens, and the specific biomarkers employed likely contributed to variability in the reported outcomes, limiting the generalizability of the findings. Second, our review focused exclusively on studies employing PEA technology, which, while offering high sensitivity and multiplexing capacity, inherently restricts the analysis to predefined panels of proteins. Third, although we identified many associated proteins across studies, the majority was investigated once or a limited number of times, weakening the robustness of the finding. This underscores the need for more evidence to support their potential clinical relevance. Fourth, the inconsistent stratification by microbiological agents precluded the possibility of assessing pathogen-specific proteomic signatures, a crucial aspect to identify targeted diagnostic or prognostic biomarkers.

Additionally, included studies adopted heterogeneous statistical approaches. The majority relied exclusively on univariable analyses, with varying degrees of methodological rigor in verifying the underlying assumptions for statistical testing. Only a minority extended their analysis through multivariable models, which can provide more reliable interpretations of the associations observed. This limited application of advanced statistics may have affected the strength and interpretability of the findings, highlighting the importance of more comprehensive analytical frameworks in future investigations.

Also, given the methodological variability across included studies, risk-of-bias assessments were used to provide a descriptive appraisal of the overall quality and limitations of the existing evidence, rather than to weigh or stratify individual findings through sensitivity analyses.

Finally, the classification of proteins as “associated” or “non-associated” was based on statistical significance as reported in the primary studies, which varied widely in terms of analytical methods. As such, this review does not provide a quantitative assessment of the magnitude, directionality, or clinical relevance of these associations, nor does it attempt formal ranking or aggregation, which would require consistent endpoints, and systematic correction for multiple testing. Consequently, absence of association in this dataset should not be interpreted as biological irrelevance.

### 4.4 Clinical implications and future directions

While affinity-based proteomic technologies like PEA hold considerable promise, several challenges must be acknowledged. Plasma protein expression levels are affected by various physiological and pathological variables, including comorbidities, pharmacological treatments, and individual host responses (Donoghue et al., 2025). These factors may confound the interpretation of proteomic signals, limiting their specificity and reproducibility in real-world settings.

Future research should prioritize the application of proteomics in clinical contexts where the burden of BIs is highest and diagnostic uncertainty is most pressing, like lower respiratory tract, bloodstream, and intra-abdominal infections, accounting for approximately 75% of BIs-related deaths worldwide (Ending preventable child deaths from pneumonia and diarrhoea by 2025: the integrated Global Action Plan for Pneumonia and Diarrhoea (GAPPD), 2013; Sartelli et al., 2017; Global report on the epidemiology and burden of sepsis: current evidence, identifying gaps and future directions, 2020). Reliable biomarkers in these high-impact settings can improve timely diagnosis and guide personalized treatments.

Among the most frequently reported proteins emerging from this review, CXCL-10, IFNG, TNFRSF9, TNFSF14, CCL-20, CXCL-8, and HGF, appeared recurrently in diverse clinical contexts and have already been implicated in host responses to bacterial pathogens in prior studies. Their repeated reporting across independent analyses supports their biological plausibility within host immune responses to BIs. Conversely, proteins that were reported as not consistently associated in this review should not be interpreted as biologically irrelevant, as their roles may be insufficiently captured by the limited PEA-based literature currently available.

Importantly, the value of proteomic technologies like PEA may lie less in the identification of single, standalone biomarkers, and more in their ability to unravel complex host-pathogen interactions and disease mechanisms (Amiri-Dashatan et al., 2018; D’Onofrio et al., 2022). By offering dynamic and functional insights into immune and inflammatory responses, proteomic analyses can contribute to a more nuanced understanding of infection pathophysiology (D’Onofrio et al., 2022; Girardis et al., 2024). This system-level perspective may support targeted diagnostics and stratified clinical management, especially in syndromes marked by immunological heterogeneity, such as sepsis (van Engelen et al., 2018; Girardis et al., 2024).

## 5 Conclusions

This scoping review highlights the emerging but still limited application of PEA-based proteomics in the study of BIs in adults. Despite its high sensitivity and multiplexing capacity, current use remains largely exploratory, with methodological variability limiting reproducibility and clinical translation.

Rather than seeking single diagnostic markers, the greatest value of proteomic approaches may lie in characterizing host responses and uncovering the biological mechanisms underlying infection and treatment outcomes. To advance the field, future research should prioritize longitudinal, well-designed, and pathogen-stratified studies in high-burden clinical settings.
