# Supplementary material for: Proximity extension assay-based targeted proteomics for biomarker discovery in adult bacterial infections
Source: Front Cell Infect Microbiol. 2026 Feb 13;16:1716483. doi: 10.3389/fcimb.2026.1716483 (PMC12945781; doi:10.3389/fcimb.2026.1716483)

***Supplementary Material***

# Search Strategy

The following search strategy was applied across MEDLINE, Embase (Elsevier), and Web of Science to identify relevant studies on the use of Olink proteomic technology in human bacterial infections:

**Search terms**: “Olink” AND “infection”

No filters or additional Boolean operators were used. The search was conducted up to February 14, 2025, and included all available records without restrictions on language, publication date, or study design.

| **Database** | **Search strategy** | **Records** |
| --- | --- | --- |
| Pubmed | “Olink” AND “Infection” | 139 |
| Embase | “Olink” AND “Infection” | 259 |
| Web of Science | “Olink” AND “Infection” | 91 |

1. **Functional Annotation of High-Priority Proteins**

To characterise the biological and functional profiles of the high-priority proteins identified in our review, we performed automated data extraction from the following publicly available databases:

- UniProt (https://www.uniprot.org/)
- DisGeNET (https://www.disgenet.org/)

Data extraction was conducted using custom R scripts developed in-house. Information was retrieved via UniProt API for protein names, functional descriptions, biological processes, and pathways, and via DisGeNET API for disease associations. The scripts queried the APIs using UniProt accession numbers or gene symbols, and the output was compiled into a structured table format. Data handling and API integration were performed using standard packages including “rvest”, “httr”, “jsonlite”, and “dplyr”. All data extraction and processing steps were performed in R (version 4.3.3). Manual verification was performed in selected cases to resolve inconsistencies or ensure data completeness. Following extraction, the annotations were manually reviewed and refined to ensure grammatical consistency and improve readability. Textual information was harmonised and, where appropriate, condensed to facilitate interpretation and enhance the clarity of tabular presentation. The resulting functional annotation dataset is provided in Table 2.

# Supplementary Figures and Tables

## Supplementary Tables

| **Variable** | **Total** | **DEPs** | **non-DEPs** | **p value** |
| --- | --- | --- | --- | --- |
| **Total Investigations °** | 1659 | 1142 (68.8%) | 517 (31.2%) | - |
| **Frequency of Analysis *** | 4 (3 - 7) | 4 (3 - 7) | 3 (1 - 4) | <0.01 |
| **Total Associations °** | 461 | 461 (100%) | 0 (0%) | - |
| **Frequency of Association *** | 1 (0 - 2) | 2 (2 - 3) | 0 (0 - 0) | - |
| **Association Proportion *** | 20 (0 - 50) | 50 (29 - 67) | 0 (0 - 0) | - |
| **Total Proteins °** | **Total**  N = 379 | **DEPs**  N = 207 | **non-DEPs**  N = 172 | **p value** |
| **Investigated Clinical Settings °** |  |  |  |  |
| *Bacterial vs Healthy* | 274 (72.3%) | 189 (91.3%) | 85 (49.4%) | <0.01 |
| *Bacterial vs Viral* | 355 (93.7%) | 196 (94.7%) | 159 (92.4%) | 0.50 |
| *Post-surgical Infectious vs Non-infectious* | 141 (37.2%) | 100 (48.3%) | 41 (23.8%) | <0.01 |
| **Associated Clinical Settings °** *[% calculated on corresponding investigated clinical settings]* |  |  |  |  |
| Bacterial vs Healthy | 178 (65.0%) | 178 (94.2%) | 0 (0%) | <0.01 |
| Bacterial vs Viral | 42 (11.8%) | 42 (21.4%) | 0 (0%) | <0.01 |
| Post-surgical Infectious vs Non-infectious | 64 (45.4%) | 64 (64.0%) | 0 (0%) | <0.01 |
| **Investigated Biological Specimens °** |  |  |  |  |
| *Plasma* | 379 (100%) | 207 (100%) | 172 (100%) | - |
| *Serum* | 92 (24.3%) | 66 (31.9%) | 26 (15.1%) | <0.01 |
| *CSF* | 92 (24.3%) | 63 (30.4%) | 29 (16.9%) | <0.01 |
| *Synovial Fluid* | 92 (24.3%) | 63 (30.4%) | 29 (16.9%) | <0.01 |
| *Prosthetic Fluid* | 92 (24.3%) | 74 (35.7%) | 18 (10.5%) | <0.01 |
| **Associated Biological Specimens °** *[% calculated on corresponding investigated biological specimens]* |  |  |  |  |
| *Plasma* | 189 (49.9%) | 189 (91.3%) | 0 (0%) | <0.01 |
| *Serum* | 6 (6.5%) | 6 (9.1%) | 0 (0%) | 0.18 |
| *CSF* | 27 (29.3%) | 27 (42.9%) | 0 (0%) | <0.01 |
| *Synovial Fluid* | 3 (3.3%) | 3 (4.8%) | 0 (0%) | 0.55 |
| *Prosthetic Fluid* | 37 (40.2%) | 37 (50.0%) | 0 (0%) | <0.01 |

*° n (%); * median (IQR);* *Acronyms: DEPs, differentially expressed proteins; CSF: cerebrospinal fluid.*

**Supplementary Table 1.** Characteristics of all the analysed proteins stratified in differentially expressed proteins (DEPs) and non-differentially expressed proteins (non-DEPs).

| **Variable** | **Total** | **DEPs** | **non-DEPs** | **p value** |
| --- | --- | --- | --- | --- |
| **Total Investigations °** | 457 (100%) | 393 (86%) | 64 (14%) |  |
| **Frequency of Analysis *** | 10 (10 - 10) | 10 (10 - 10) | 10 (10 - 10) | 0.91 |
| **Total Associations °** | 130 (100%) | 130 (100%) | 0 (0%) | - |
| **Frequency of Association *** | 3 (2 - 4) | 3 (2.25 - 4.75) | 0 (0 - 0) | - |
| **Association Proportion *** | 30 (20 - 40) | 30 (20.4 - 40) | 0 (0 - 0) | - |
| **Total Proteins °** | **Total**  N = 44 | **DEPs**  N = 38 | **non-DEPs**  N = 6 | **p value** |
| **Investigated Clinical Settings °** |  |  |  |  |
| *Bacterial vs Healthy* | 44 (100%) | 38 (100%) | 6 (100%) | - |
| *Bacterial vs Viral* | 44 (100%) | 38 (100%) | 6 (100%) | - |
| *Post-surgical Infectious vs Non-infectious* | 44 (100%) | 38 (100%) | 6 (100%) | - |
| **Associated Clinical Settings °** *[% calculated on corresponding investigated clinical settings]* |  |  |  |  |
| *Bacterial vs Healthy* | 36 (81.8%) | 36 (94.7%) | 0 (0%) | <0.01 |
| *Bacterial vs Viral* | 19 (43.2%) | 19 (50.0%) | 0 (0%) | 0.03 |
| *Post-surgical Infectious vs Non-infectious* | 26 (59.1%) | 26 (68.4%) | 0 (0%) | <0.01 |
| **Investigated Biological Specimens °** |  |  |  |  |
| *Plasma* | 44 (100%) | 38 (100%) | 6 (100%) | - |
| *Serum* | 4 (9.1%) | 3 (7.9%) | 1 (16.7%) | 0.46 |
| *CSF* | 43 (97.7%) | 37 (97.4%) | 6 (100%) | 1.00 |
| *Synovial Fluid* | 43 (97.7%) | 37 (97.4%) | 6 (100%) | 1.00 |
| *Prosthetic Fluid* | 44 (100%) | 38 (100%) | 6 (100%) | - |
| **Associated Biological Specimens °** *[% calculated on corresponding investigated biological specimens]* |  |  |  |  |
| *Plasma* | 37 (84.1%) | 37 (97.4%) | 0 (0%) | <0.01 |
| *Serum* | 0 (0%) | 0 (0%) | 0 (0%) | - |
| *CSF* | 11 (25.6%) | 11 (29.7%) | 0 (0%) | 0.31 |
| *Synovial Fluid* | 2 (4.7%) | 2 (5.4%) | 0 (0%) | 1.00 |
| *Prosthetic Fluid* | 19 (43.2%) | 19 (50%) | 0 (0%) | 0.03 |

*° n (%); * median (IQR);* *Acronyms: DEP, differentially expressed proteins; CSF: cerebrospinal fluid.*

**Supplementary Table 2.** Characteristics of the proteins analysed 10 or more times stratified in differentially expressed proteins (DEPs) and non-differentially expressed proteins (non-DEPs).

| **Category** | **0-24%** | **25-49%** | **50-74%** | **75-100%** | **Row Total** |
| --- | --- | --- | --- | --- | --- |
| ***Non-associated*** | 6 | 0 | 0 | 0 | 6 (14%) |
| ***Bacterial vs Healthy only*** | 7 | 0 | 0 | 0 | 7 (16%) |
| ***Bacterial vs Viral only*** | 1 | 0 | 0 | 0 | 1 (2%) |
| ***Post-surgical infectious vs non-infectious only*** | 1 | 0 | 0 | 0 | 1 (2%) |
| ***Bacterial vs Healthy + Bacterial vs Viral*** | 0 | 3 | 1 | 0 | 4 (9%) |
| ***Bacterial vs Healthy + Post-surgical infectious vs non-infectious*** | 2 | 8 | 1 | 0 | 11 (25%) |
| ***Bacterial vs Viral + Post-surgical infectious vs non-infectious*** | 0 | 0 | 0 | 0 | 0 (0%) |
| ***Bacterial vs Healthy + Bacterial vs Viral + Post-surgical infectious vs non-infectious*** | 0 | 7 | 7 | 0 | 14 (32%) |
| ***Total Proteins*** | 17 | 18 | 9 | 0 | 44 (100%) |

**Supplementary Table 3.** Distribution of the proteins analysed >10 times, shown by association proportion and clinical comparison type. All listed proteins were evaluated in the context of all three clinical comparisons included in the review.

## Supplementary Figures
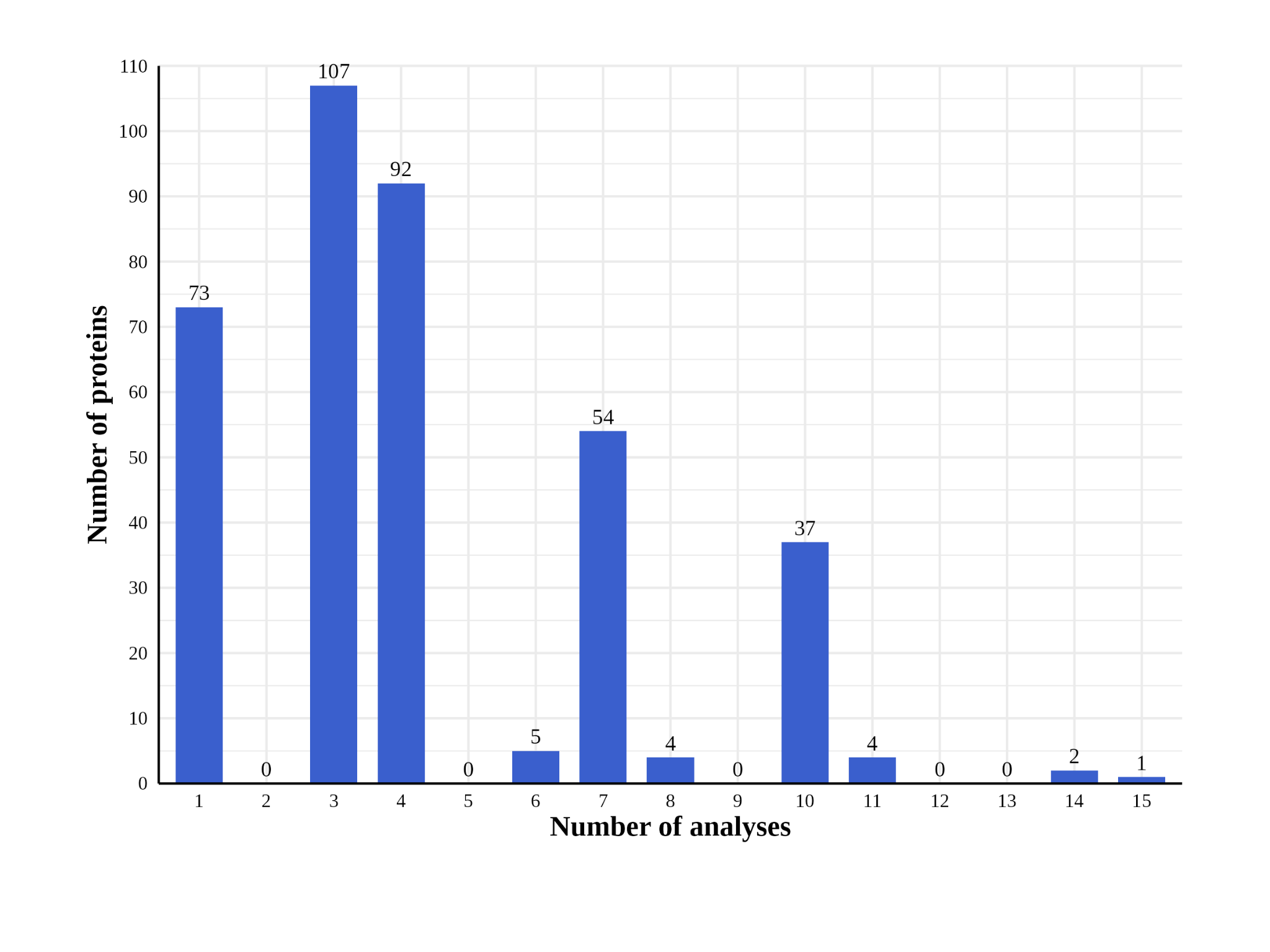


**Supplementary Figure 1.** Distribution of included proteins by number of investigations.

**Supplementary Figure 2.** Distribution of proteins across all types of comparisons categorised by the number of times they were investigated. P-values obtained from McNemar tests are reported for each category, with statistical significance indicated where appropriate.
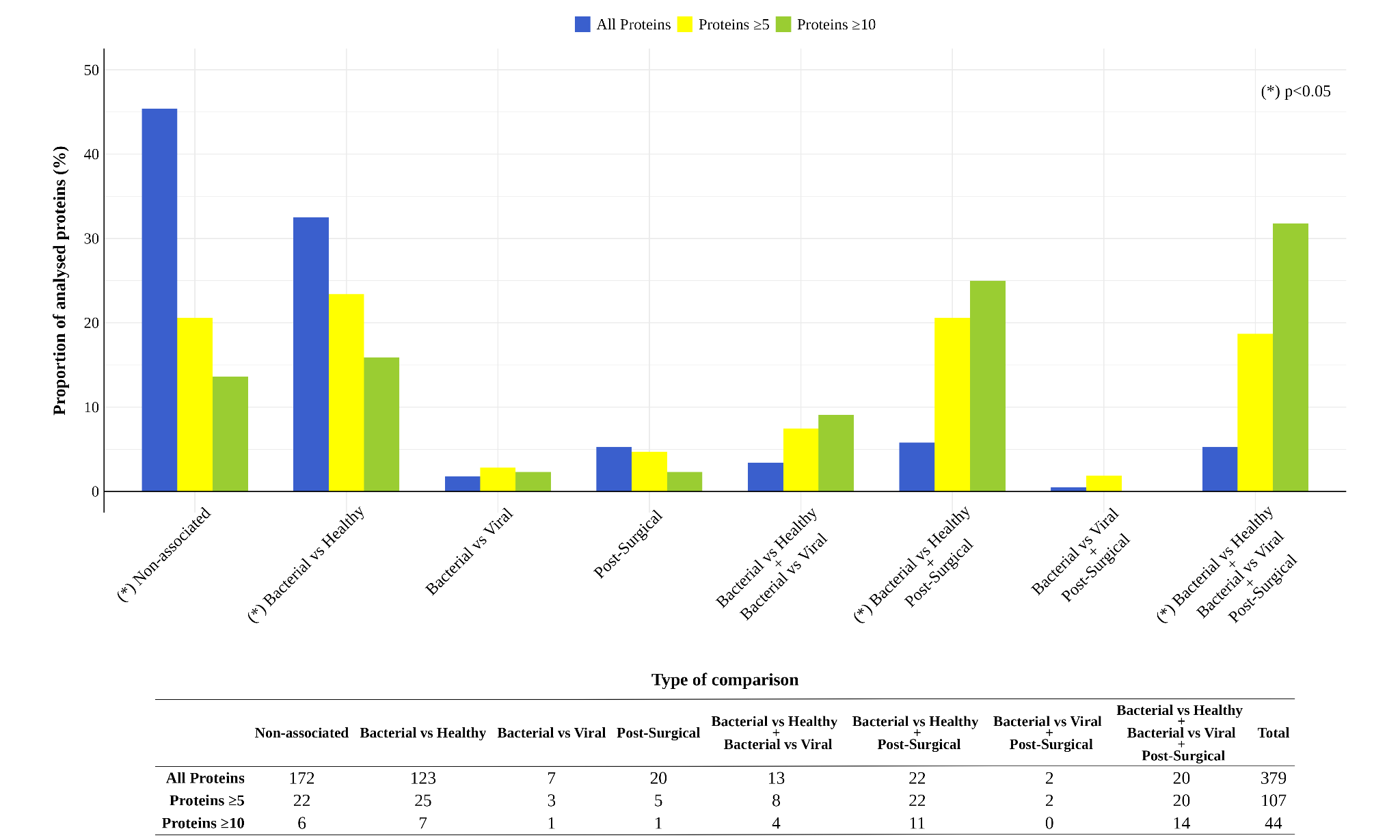


**Supplementary Figure 3.** Risk of bias assessment of the studies included in the review. Evaluated using an adapted version of the ROBINS-E tool, focusing on case/comparator selection, group comparability, and outcome assessment.
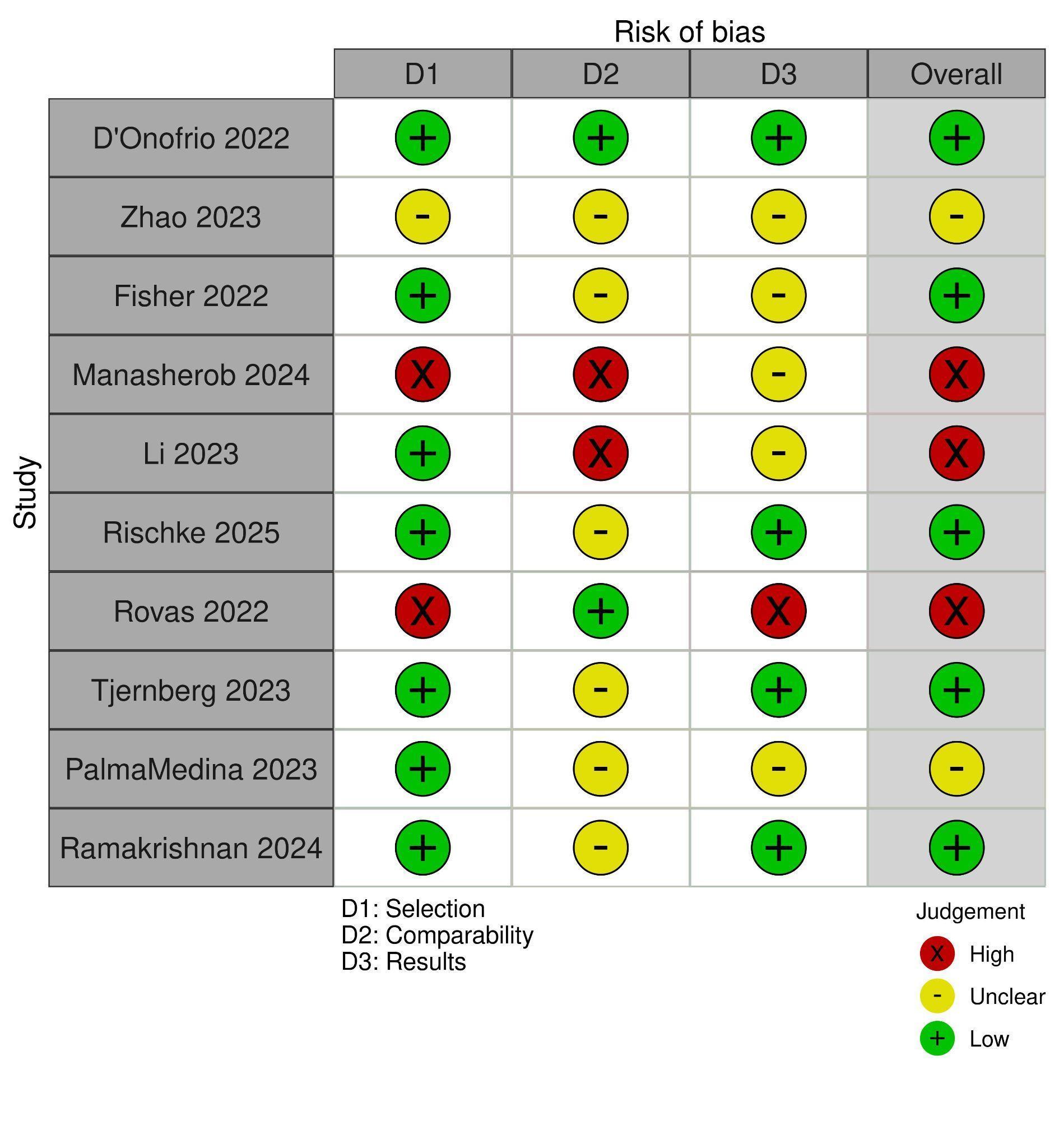

Supplement: Supplementary file 1 [file Table1.docx]
